# Psychological distress among Iranian health-care providers exposed to coronavirus disease 2019 (COVID-19): a qualitative study

**DOI:** 10.1186/s12888-020-02889-2

**Published:** 2020-10-07

**Authors:** Azizeh Alizadeh, Hamid Reza Khankeh, Mohammad Barati, Yazdan Ahmadi, Arash Hadian, Maryam Azizi

**Affiliations:** 1 NEZAJA Health Department, Assistance of Mental Health, Tehran, IR Iran; 2 University of Social Welfare & Rehabilitation Sciences, Tehran, Iran; 3 Department of Clinical Science and Education, Karolinska Institutet, Stockholm, Sweden; 4 Infectious Diseases Research Center, AJA University of Medical Sciences, Tehran, IR Iran; 5 Faculty of Nursing, AJA University of Medical Sciences, Tehran, IR Iran; 6 Department of Clinical Psychology Hajar Hospital, Tehran, IR Iran

**Keywords:** Psychological distress, Health-care providers, COVID − 19, Iran, Qualitative study

## Abstract

**Background:**

Novel corona virus, named COVID-19, has spread rapidly to other countries like Italy, Iran and South Korea and affected all people, especially health-care providers. Therefore, due to the rapid spread of the disease in Iran, the aim of the present study was to explore psychological distress experienced by Iranian health-care providers in the first few weeks of the corona virus outbreak.

**Methods:**

The present qualitative study was conducted on 18 Iranian health-care providers exposed to COVID − 19 using a content analysis method. Purposeful sampling was used to select the participants and continued until data saturation was reached. Data were collected using semi-structured interviews and then the qualitative data were analyzed through direct content analysis.

**Results:**

By analyzing 236 primary codes, two main categories were extracted from the experiences of health-care providers during corona virus outbreak. The first category included Occupational demands with three sub-categories: nature of illness, Organizational demands and social demands. The second category was Supportive resources included personal support and social support.

**Conclusions:**

The results of this study found that there were some barriers and challenges to medical personnel exposed to COVID-19 that caused psychological distress. Some of these problems related to the nature of illness, others related to social and organizational demands and some of supportive resources buffer the relationship between occupational demands and psychological distress.

## Background

In the end of December 2019, a novel corona virus named COVID-19 by World Health Organization (WHO), was reported in the Chinese city of Wuhan for the first time. This virus has spread rapidly to other countries including Italy, Iran and South Korea [1, 2].

According to data released by the Ministry of Health of the Islamic Republic of Iran, the first confirmed cases of COVID-19 infections were reported on 19 February 2020 in Qom Province. Later that day, two people who had tested positive had died. In the following days, the disease spread to most of the provinces of Iran. Iranian health care providers are fighting against this life-threatening virus on the front lines of corona-specific hospitals. Unfortunately, this fatal new virus has infected medical workers, with about 150 deaths in Iran [3].

COVID-19 outbreak has a significant psychological impact on various levels of society. Hence, high levels of anxiety, depression, stress, fear, boredom, loneliness, uncertainty, post-traumatic stress symptoms, confusion, anger and stigma, which are the signs of psychological distress, are significant in patients with confirmed or suspected COVID − 19 during this epidemic because they are in quarantine [4–8].

Psychological distress is an unpleasant objective state of depression and anxiety that has physical and emotional manifestations and is an indicator of psychological problems that are examined in research and clinical collections with psychosocial and behavioral symptoms related to anxiety and depression [9]. Of course, psychological distress is not specific to a particular pathology. These conditions include anxiety, depression, mood swings, decreased intellectual ability, sleep problem, absenteeism, etc. [10]. Also, psychological distress is the emotional suffering caused by a real or perceived physical or psychological threat that a person is experiencing. Psychological distress as a key indicator describes a person’s emotional problems and psychological responses to adaptation to the environment and negatively affects a person’s job capacity, family life, and well-being. These psychological distress increase during COVID − 19 outbreak [6].

In the health area, nursing stands out as one of the most exhausting professions owing to different circumstances in professional practice causing physical and emotional exhaustion. The nurse is expected to perform patient care with patience and empathy, all in a highly stressful environment, with few resources and an excessive workload, thereby requiring nurses to find a balance between these factors that interfere in their working life (Medeiros de Oliveira, 2019). This psychological distress may be reinforced by increasing the symptoms of infection and the side effects of the treatment [6].

**Fig. 1:**
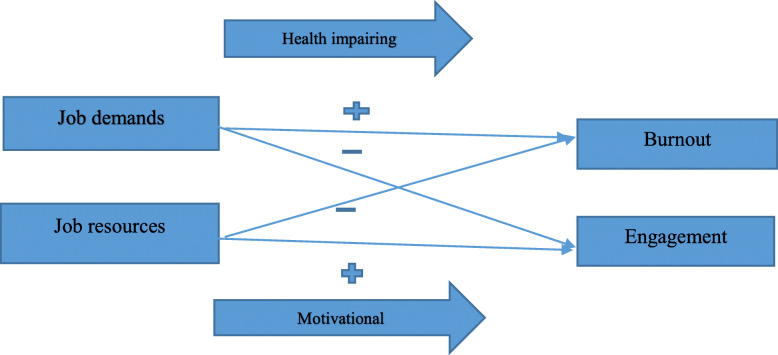
Key assumptions of the JD-R model

Following an increasing number of confirmed and suspected cases with COVID-19; medical workers have suffered from enormous pressure due to physical and psychological stress, including high job demand and lack of resources, isolation, inadequate protection and high risk of infection, frustration, exhaustion and discrimination [2, 11]. According to Job Demand Resource Model (JD-R), demands are any physical, social, psychological or organizational aspects of the job that require the employee to continually engage in physical or mental effort and resources are physical, social, psychological or organizational aspects of the job that play an intrinsic motivational role. The JD-R is comprehensive, explaining how job demands and resources have unique and multiplicative effects on job burnout and work engagement [12]. This model assumes two key underlying pathways. Firstly, Job demands; a health impairment pathway, whereby badly designed jobs or chronic job demands activate an energy depletion process which can lead to negative outcomes. Secondly, Job resources; whereby carry motivational potential leading to positive outcomes [13] (Fig. 1).

**Fig. 2:**
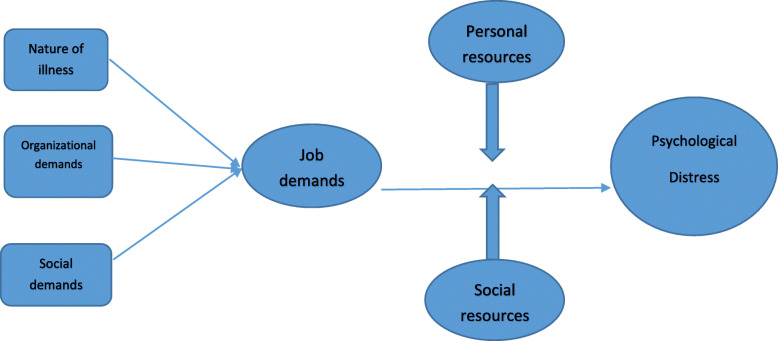
Factors of psychological distress in Iranian health-care providers in the first two weeks of the corona virus outbreak using the JD-R model

The COVID-19 pandemic appears to have increased demand and reduced resources. Therefore, medical workers are vulnerable to psychological distress and other mental health symptoms such as depression, anxiety, insomnia, denial, anger and fear of contagion and spreading the virus to others during the COVID-19 outbreak [2, 8, 11]. These mental health problems affect the staff’s mental well-being and decision-making ability to control the COVID-19 pandemic, so maintaining mental health of clinical medical workers is important and urgently needed for controlling infectious diseases [8, 11].

Since no qualitative studies have been conducted in this area so far, and the process of health care provider services in the face of COVID-19 outbreak is a phenomenon that can be explored from participants’ experiences, this content analysis study was conducted to analyze data from observations and interviews with front line medical workers and to explore factors influencing psychological distress in Iranian health care workers who are fighting with COVID-19 infection in order to inform healthcare decision-makers a timely understanding of mental health status for providing proper interventions.

## Methods

### Study design

This is a qualitative study of psychological distress among health-care providers who worked directly in the medical wards of Tehran hospitals for patients with COVID-19, in the first few weeks of the corona virus outbreak. This was an inductive qualitative content analysis study of psychological distress among health-care personnel who were directly exposed to patients with COVID-19 in Tehran. The researchers performed an in-depth direct analysis of nurses’ and doctors’ experiences. The results were presented as codes, subcategories, and categories using an inductive approach. Finally, the results were organized into the framework of the JD-R model [13].

### Participants and study setting

Participants were selected through the purposive sampling method from several hospitals that were engaged in treatment of patients with COVID-19 during the outbreak of the corona virus. Selection of samples was based on the objective of the study, and in light of the research question, study samples included groups of health care providers including practical nurse, nurses and doctors. Eighteen participants, including nurses and doctors exposed to COVID-19, were selected based on the question and objective of the study. Interviews with personnel were done at their workplace. Each interview lasted about 30–45 min and it was conducted at their workplace with their consent. The endpoint for sample selection was reaching data saturation. At this point, additional information obtained from the three final interviews did not vary the categorizations.

### Data analysis

First, interviewer proposed some general questions to start the interviews such as “Please explain one of your assignments”. Data were collected through semi-structured interviews from February 29 to March 20, 2020. Interviewers took place both in private and public facilities in Tehran province, which exposed to patients with COVID-19 according to purposive sampling based on a convenience sampling approach. Some of these hospitals were entirely dedicated to care patients with COVID-19, other hospitals just specified a few wards for caring these patients, but those Health-Care Providers who exposed to care patients with COVID-19 selected as participants. Interviews were conducted in Farsi language by the authors who were experienced in psychological interviews and qualitative research, and then they were analyzed in the local language, after that, translated in English language by authors and edited by bilingual editor.

Interviews with personnel started with their experience about related psychological distress, and according to the interview guidelines, general open-ended questions were asked saying “when you were taking care of patients with COVID-19, what factors bothered you in the first few weeks of the corona virus outbreak? Or what things were your concerns and anxieties in the first few weeks of the corona virus outbreak? Then, depending on the context of the responses, the interviewer continued with exploratory questions “can you please give an example?” or “could you explain more?” to clarify concepts for the researcher and participants. Since certain issues could be beyond the scope of the interview, respondents were given the opportunity for an informal conversation at the end of the interview by asking them “would you like to add anything else.

After each interview, audio files were listened to several times and verbatim transcriptions were prepared using MAXQDA. Each entire interview was considered as an analysis unit. The transcribed script was read several times to become familiar with the context, after which, the meaning units were identified. Then, the condensed meaning units were abstracted and labeled with a code (Table 1), which were then compared based on differences and similarities and sorted into sub-categories and categories [14] (Table 2). Finally, the results were organized into the framework of JD-R model. According to JD-R model, a high job demand and low job resources can lead to burnout and job pressure [15].

**Table 1:** Examples of meaning units, codes and Sub-categories from content analysis of the health-care provider’s experiences

Meaning unit	Code	Sub-categories
- It was news that we heard about the deaths of doctors, especially the news that emphasized the deaths of colleagues and patients, and especially the deaths of doctors in different cities, have a very bad effect on us.	fear of more mortality	Nature of illness
-In the early days, we did not have any equipment at all. The simplest ones were gloves and a mask, which I took from home with some of my colleagues. Face masks (the FSP and N95 masks), gloves and other protective equipment were rationed to avoid shortages. They did not give us more than two dozen of ordinary masks, and we had to buy or make our own masks.	Shortage	Organizational demands
The bad attitude of the people scares me	stigma	Social support

**Table 2:** Examples of codes, sub-categories and categories from content analysis of participants’ experiences

Theme	Psychological distress	
Categories	Occupational demands	Supportive resources
Sub-categories	Nature of illness	Social support
Codes	fear of more outbreaks I am afraid of being a carrier of the disease and spreading it to my family and children	Appreciation of nurses It made me happy when I saw the appreciation of officials and people from medical staff and nurses on TV

Several strategies were used to ensure the credibility of data. In this regard, data gathering lasted about 1 month and researchers were deeply oriented to data and atmosphere of the field during this time. To ensure the credibility of the data, some strategies including Peer review (data and interpretation of data were checked by other researchers), and Member checking (data rechecked by participants and our interpretations from data were reviewed and confirmed by them) were used.

### Ethical considerations

The Ethics Committee of the Aja University of Medical Sciences approved this study. Target individuals were informed about the purpose of the study and their right to withdraw from the study at any point. All the participants were asked to sign the informed consent form and, their anonymity and confidentiality was ensured.

## Results

The participants of the study were 18 people, 10 women and 8 men. The participants ranged in age from 24 to 42 years, including 6 physicians and 12 nurses, of whom 13 were married, and 5 were single. In the present study, participants fall into two general categories that contributed to the psychological distress of health care personnel. The first category included Occupational demands with three sub-categories: nature of illness, Organizational demands and social demands. The second category was resources included personal support and social support (Fig. 2). Examples of meaning units, codes and Sub-categories from content analysis of the health-care provider’s experiences described in Table 1. The data from interviews showed 236 open codes in 5 sub-categories and 2 categories were obtained in this context. Categories and subcategories have been shown in Table 3.

**Table 3:** Coding of participants’ experiences

Codes	Subcategories	Categories	Main theme
work overload, wearing protective clothing, ambiguity about the disease, quarantine, fear of more outbreaks, fear of being at work, sleep problems, fear of more mortality	Nature of illness	Occupational demands	Psychological distress
Injustice Economic problems Management’s problem shortages	Organizational demands		
Stigma Media Ignoring quarantine	Social demands		
Problem-oriented coping, compassion satisfaction, spirituality, personality traits	Personal supports	Supportive Resources	
teamwork, family and friend’s supports appreciation of nurses	Social supports		

### First category: occupational demands

#### Nature of the disease

The staff noted that the causes of the psychological distress, caused by the nature of the disease, included work overload, wearing protective clothing, ambiguity about the disease, quarantine, repetitive work, fear more outbreaks, fear of being at work, sleep problems, and fear of more mortality.

Regarding workload, some interviewees cited high work pressure, hard work, difficult and stressful working conditions, and lack of vacations. For example, participant No. 1 said: “If I want to talk about the working conditions of nurses before corona, the working conditions were very difficult; now corona has been added to it, and the burden of stress and extra and new work has made the situation much worse”.

Difficulties of wearing protective clothing are another concept that most employees declared in sub-category named “the nature of the disease”. For example, participant No. 15 said: “Wearing a mask and gloves and also using a face shield for several hours are very difficult because it cannot be tolerated for an hour, but you have to endure it in six hours and you cannot even meet your basic needs”.

Another concept that causes discomfort is the lack of definitive treatment for the disease. Participant No. 15 said: “It causes us to have a lot of mortality here. The patient comes with a lung infection and we cannot do anything here. Those whose lungs are completely involved. We do not have the right treatment for them. The treatments are supportive to see how long the body lasts. On the other hand, Participant No. 8 said: “Every day they gave me a new protocol, every day they said to do this, tomorrow they said this is wrong, do that.” These issues were confusing.

Another concept in the nature of the disease was “quarantine”. For example, participant No. 8 said: “We cannot visit our families and public places like shopping centers, leisure venues, etc. Well, sometimes walking down the shopping gives people peace of mind that we are deprived.” Or participant No. 2 said, “We cannot see our family. We have been in quarantine for about a month and a half now; we cannot even kiss our family members and friends.”

The next concept in the nature of the disease is “repetitive work”. As participant, No. 8 said: “The repetition of days and things have really made the situation difficult. The monotonous repetition of daily work had a negative effect on our mind.”

A concept of this section was the fear of attendance at work. Participant No. 9 said: “In the first days, there was a great fear among doctors and nurses which we all wanted to avoid accepting patients in hospital.” Alternatively, the participant No. 2 said: “the night we wanted to back work, we wished and asked God never back to the hospital.”

“Fear of infection” is another concept of “the nature of the disease”. In this regard, Participant No. 11 said: “I have been most concerned about my family since this period began. Anyway, every time I come here, the environment is polluted and there is a possibility that I will be the carrier of this disease.” Also, participant No. 9 said: “The fear of getting sick was both for myself and for those around me and my family, I have the same fear right now, and this has made me nervous.”

“Fear of more outbreaks” is another concept related to “the nature of the disease”. Participant No. 1 said, “I am really worried about more outbreak.”

The next concept is the “fear of more mortality”. In this regard, participant No. 16 said: “The death of patients and colleagues, especially medical colleagues in different cities, had a very bad effect on us.”

“Sleep problems” are another concept of “the nature of the disease”. As participant No. 9 said, “I woke up frequently at night and I was stressed and dreaming of the dead.”

#### Organizational demands

“Injustice” is one of the organizational demands, as the participant No. 8, said: “I think they are abusing us, indirectly said that this is your job and you have to do it”. Additionally, there is mistrust. In this regard, participant No.3 said: “Our most concern is that after the crisis, nurses would be forgotten again.” At this level, “the provision of compulsory services” was another issue that some employees pointed out, for example, the participant No. 10 said: “The fact that we had to work under duress and pressure bothered me and I was annoyed why I had to be forced.”

“Economic problems” were another distress at this level. For example, participant No. 8 said: “economic difficulties caused by sanctions in our country was one of my concern because the health system cannot meet the people needs well and this disease get out of control.” “It is very annoying that our arrears that should have been paid years and months ago have not been paid.

Another concept at this level was “Management’s problems” included Lack of transparency of managers, manager’s anxiety, lack of efficient management and misconduct of managers. Participant No. 6 said: “Lack of transparency of decisions taken.” The same participant said: “One of the main problems that bothered many staff was manager’s anxiety. At first, managers should be able to control their stress and should not suffer anyone, but unfortunately our boss and managers were in crisis themselves and could not lead the crisis.”

Participant No. 8 said: “Another thing that bothered me was mismanagement at different levels”. “misconduct of managers” as the participant No. 6 said: “very aggressive behavior of one of my managers made me very upset that all the staff had felt these behaviors and regretted the behavior of the senior managers”.

In this category, participants discussed shortages in equipment and manpower that caused distress. For example, participant No. 9 said: “We saw how much stress our colleagues endured to get the protective equipment of masks and gloves in the first days of corona virus outbreak.” In addition, participant No. 5 said: “There has been a lack of personal protective equipment, including masks, clothes, gloves, etc., that nearly all hospitals face with this problem.”

Regarding deficiency and fatigue of human resources, participant No. 4 said: “There was not enough infectious and internal specialists and nurses in the center.” There were a lack of special equipment, as participant No. 6 said, “COVID-19 test was not taken from all of the personnel; it was taken if they had symptoms.” Improper nutrition was another issue raised in this category. Participant No. 15 said: “Another problem is that the children complained of poor nutrition. Some days the quality of food (dinner or lunch) they gave the staff here was not good.” Or participant No. 2 said: “We had problems in transferring to our hospital; Due to quarantine, there were no cars.”

#### Social demands

Regarding “stigma” on the class of demands of the community, the participant No. 9 said: “Once an ambulance driver did not allow us to sit in the front seat, because he did not want to get sick.” Or participant No. 2 said: “I am worried that the society will not accept us anymore. We even wanted to extend the rent of the house last month but they said that we are nurses and they did not accept us.”

The Media coverage was listed in the Social demands to note that Media tend to overemphasize negative news. As participant number 4, said: “we all receive negative news, ambiguities and rumors about this disease”; also, participant No. 5 said: “Rumors and exaggeration of virtual networks and news bother me a lot.”

The people’s lack of attention to health and quarantine orders is one of the things that the participants called distress. For example, participant No. 11 said: “In this situation, I am afraid of people’s negligence; some people are very careless. We see some who have no symptoms but come to the hospital to get a simple medicine, or some have symptoms but we see that they have no mask.”

### Second category: supportive resources

#### Personal resources

In this category, participants talked about effective Personal resources for reducing their distress during corona virus outbreak. These codes were included 5 categories: problem-oriented coping, compassion satisfaction, spirituality, personality traits and social support.

In the problem-oriented concept, concepts such as following the instructions, doing favorite things, gain valid information, lack of attention to news, and maintaining morale were obtained. For example, regarding the concept of following protocols, participant No. 4 said: “We provided the safety equipment for ourselves. We suggest what we should do. We emphasize hand washing, keep repeating the use of medical equipment and keep our distance from the patient. We try to do more in this process.”

Participants No.3, regarding gaining a valid information concept, said: “We were searching, reading up-to-date articles, reading WHO site, and I was doing some research myself on the COVID-19. Now, I’m sending this article to Lancet Magazine, which I hope will be accepted. We tried to send each other up-to-date content, and this made it possible for us to reduce some of our stress and nervous problems.”

“Doing favorite things” was another individual resource, which participant No.4 noted: “These days, I try to read more books in my spare time. I want to watch comedy movies and listen to my favorite songs.”

Based on the participants’ accounts of their experiences, compassion satisfaction with concepts such as empathy with the patient, the joy of improving patients’ health, interest in people, and commitment to the patient may act as one of the effective individual resources for reducing stress.

Some participants mentioned empathy with the patient. As participant, No. 12 said: “Mostly, patients’ anxiety and worries bothered me, and made me very sad.”

Some participants mentioned that they feel satisfied with the patient’s recovery. As participant, No. 5 said: “If a patient is discharged and his family take him to leave the hospital, I will be fine.” Also, participant No. 12 said: “I felt good for helping, and I did not have any worries or fears. I enjoyed it more when I saw someone happy because I helped him/her. I felt satisfied.”

Spirituality was another category, including strong faith, and hope to God’s grace and pray. Some participants, such as the participant No. 4, regarding spirituality, said: “I work in a place where, in return for the health of that patient, a smile comes to their lips makes me happy. I do this to please God and I hope this feeling will never go away.”

Another concepts included personality traits, including high self-confidence, challenging interest, realism, sense of humor, high adaptability, hope, courage, strong thinking, adherence to ethics, relax, and flexibility.

Participant No.14 noted about interest in the challenge as a personality trait and said: “What makes my situation more bearable is that I really like the challenge and I hate working behind the desk and doing a routine job.” Participant No. 11 noted a high compliance capability with these statements: “I adapt myself very quickly to each situation. Of course, sometimes I was grumbling, but it did not make me very upset, or I did not care about others’ bad behavior.” In this regard, for ‘humor’, participant No. 3 said: “at work, we try to do fun to make the space happy.”

### Social resources

Finally, in the social support category, we found concepts such as teamwork, family and friend’s supports, and appreciation of nurses. Participant No. 13 noted: “When I saw people thanking me or saying we would like to take photo with you, and when I saw people’s good reactions and awareness about our work’s difficulties, it made me very happy.”

## Discussion

Based on the findings of the current study, two groups of factors, including occupational demands (nature of illness, social and organizational demands) cause psychological distress and supportive resources (personal and social resources) buffer the relationship between psychological distress and demand among health-care personnel who are directly involved in the treatment of patients with COVID-19 in Iran. Although the present study has an inductive approach in which concepts are extracted from qualitative data, it can be placed in a structured and well-known model in the field of job strain named JD-R model. This model has two constructs, job demands and job resources, which are against each other, as job resources with injecting energy to system can reduce job demands and its related costs [15].

Based on the results of this study, medical personnel exposed to COVID-19 experience specific conditions with different demands (nature of illness, social and organizational demands), which put a lot of pressure on them. The unknown nature of the disease, lack of definitive treatment, high risk of infection and the fear of spreading the disease to others and psychological demands caused by it have caused severe distress in Iranian health-care personnel. Also, medical staff in South and Southeast Asia countries, such as Italy, had similar problems due to high work pressure and lack of protective devices. Studies have revealed psychological impacts of this life-threating virus on people, especially medical staff. As in Italy, two infected nurses committed suicide due to fear of spreading COVID-19 to patients. It is possible that fear and anxiety of falling sick or dying, and helplessness will drive an increased suicide rates in 2020 [16].

One of the nurses’ concerns about the findings was the reaction of society as not accepting and another factor causing distress was media news. COVID-19 epidemic leads to an increased level of public anxiety and worries such as fear of stigma, not accepting in society and media news, which are the factors that the participants in the present study mentioned. Moreover, these mentioned factors have a negative effect on disease control. In addition, media with sudden and continuous stream of sensational news, which has highlighted COVID-19 as a unique threat, has added to panic, stress, and hysteria [3, 4, 17, 18]

Another staff’s demand that leads to psychological distress is inefficient leadership. According to the theory, engaging leaders included 1) Inspiring their followers, 2) Strengthening their followers, and 3) Connecting their followers. By inspiring, strengthening and connecting leadership, the fulfillment of staff’s basic psychological needs would promote. It appears that engaging leadership has an indirect effect on prevention of burnout and increasing engagement by reducing demands and increasing resources, respectively. Strengthening leaders provide their followers with work resources and development resources and monitor their qualitative and quantitative job demands. Finally, engaging leaders connect their followers by providing them social resources [5, 19]. Having an engaging management can be very effective in motivating, uniting and reducing employees’ distress during the COVID-19 outbreak.

Although the leadership and efficient management factors in our study were considered as a demand, that has led to increased workload and psychological distress, but we faced with effective management factors, which were reported by the participants. This side of leadership improved conditions by increasing employee efficiency. Inspiring managers with providing effective and timely feedback, paying attention to employees’ positive personality traits and appreciating them for their sacrifices, as effective factors, can help staff to expand their perspectives and plans which in turn leads to increased self-confidence, autonomy and a sense of empowerment in caring patients in these difficult and exhausting conditions of corona epidemic. Finally, managers with strengthening teamwork and unity in medical staff, who are in quarantine and forced to cut face-to-face relationships with their family help them to get rich social support resources to deal with corona virus.

Another aspect of the JD-R model is resources; shortages of essential and primary self-protection equipment against the virus like masks, gloves, and gown and inefficient manpower in the present study imposes terrible distress on employees, especially in early days of the virus outbreak. Based on studies, health needs created by the corona virus pandemic go well beyond the capacity of U.S. hospitals. Emerging viral pandemics can place extraordinary and sustained demands on public health and health systems and on providers of essential community services. As, lack of special masks for nurses in the United States made them to reuse single use in the United States, or same conditions in Italy made physicians to dedicate care beds and ventilators to patients who can benefit most from treatment [6, 12].

.It is clear that COVID-19 has endangered the satisfaction of psychological needs introduced as a crucial mediator and underlying levels in the JD-R model, which motivate an individual’s behavior and avoid maladaptation. Three basic psychological needs, namely autonomy, competence, and relatedness should be satisfied to foster an individual’s well-being and job performance in the work and organizational context [7, 20]. Based on our findings, in corona outbreak, the need for staff autonomy is disrupted easily by the compulsory services they have to provide, the cancellation of vacations, additional and unintended shifts, and initial deficiencies beyond one’s control. The need for staff competency was also affected in this situation. Experienced employees felt ineffective due to the unknown nature of the disease and the ambiguities surrounding treatment with no definitive cure for the disease. According to JD-R model, another need is relatedness. Staff were forced to stay away from their families in quarantine conditions. Due to fear of carrying disease, they could not see or hug their children. They had to distance themselves from the people who had lived with them for years and could not visit their friends. Therefore, this situation dominated all the psychological needs that employees needed for motivation and good performance.

Despite all lack of resources and demands caused by corona virus epidemic, findings from the present study pointed to sources that could buffer the effects of different types of distress. One of the buffering factors between job demands and job resources was personal resources defined as “positive self-evaluations that are linked to resiliency and refer to individual’s sense of ability to control and impact upon their environment successfully” such as self-efficacy, optimism, and self-esteem [5, 19]. Participants emphasized individual resources for improving the situation, including personality traits, spirituality, compassion satisfaction and active coping strategy; without the presence of these personal resources, the various job demands can easily create negativity leading to the development of signs associated with depression, job strain and symptoms that are similar to post-traumatic stress disorder [8, 21].

One of the personal resources is personality traits (e.g., hope, high self-esteem, humor). Emotional exhaustion and depersonalization are positively correlated with neuroticism and negatively correlated with agreeableness, conscientiousness, extraversion and openness. Personal accomplishment has a negative correlation with neuroticism and negative correlations with agreeableness, conscientiousness, extraversion and openness. Finally, emotional exhaustion and depersonalization have a positive correlation with anxiety and depression, while personal accomplishment has a negative correlation with anxiety and depression [9, 22] nurses are more likely to develop high levels of burnout if they present high levels of neuroticism and low levels of friendliness and responsibility [10, 23].

Another personal resource mentioned by the majority of the participants was concerns about patients (e.g. empathy with the patient, happiness from the patient’s recovery, etc.), which in the JD-R model named as compassion satisfaction. It is defined as the caregivers feeling from helping those who have experienced a disaster. Compassion satisfaction protects professional caregivers from the negative aspects of helping others with highlighting the positive aspects. Researchers declared that higher levels of compassion satisfaction were positively associated with higher self-efficacy beliefs, sense of community and constructive coping strategies. So, it can potentially have positive ramifications on individual and organizational outcomes such as buffering the relationship between high job demands and experiences of job strain. More specifically, when compassion satisfaction was high, the effect of role overload on job strain was significantly reduced [8, 21].

Spirituality (e.g. strong faith) is another personal resource in the present study. Increased spiritual well-being might reduce burnout among intensive care unit nurses [11, 24]. In this regard, Busby (2019) mentioned the role of spirituality and faith to God as powerful mechanism to control stress, burnout, and the disenchantment during struggles situations [12, 25].

Finally, coping mechanisms are necessary when dealing with stress and accompanying stressors and classified as problem-based and emotion-based [26]. Several studies highlighted problem-solving approach as the most common coping behaviors in nursing students while the avoidance approach as the least utilized coping behaviors. Problem-based coping ways are known to be beneficial to students’ learning, clinical performance and well-being, while emotion based coping ways were found to be harmful to their health [13, 26].

Another factor cited as a mediator in reducing staff distress is social support. Moderating effect of social support on the relationship between workload and burnout dimensions like emotional exhaustion and disengagement were investigated. The results of the present study showed social support received by nurses in workplace from supervisors and coworkers was found to play a fundamental role in preventing the burnout symptoms [27]. People’s appreciation of nurses, doctors and other hospital staff, in the media by calling them “health defenders” were factors in encouraging health care providers who struggled with this life-threatening disease in hospitals.

Two key interaction effects between job demands and resources, Firstly, there is the assumption that job resources buffer the negative impact of job demands on burnout. For example, high levels of job resources have been shown to reduce the relationship between job demands and work home interference. Secondly and more recently, the coping hypothesis proposes that job resources are particularly salient in conditions of high job demands because individuals draw on resources at times of stress as a means of coping. It is thought that for job resources to be the most effective at creating a motivational component, the individual must be presented with a demand that is perceived as a positive challenge [13].

In order to reduce the symptoms of psychological distress and better management of conditions in COVID-19 outbreak, strategies such as increasing facilities and resources, foster accountable management with good relationships with employees, and fairness in distribution of duties and resources, adequate and timely payments, and considering specific psychological and physical conditions of each employee are recommended also, strategies like preparation rooms with adequate facilities like proper nutrition, pleasant smells, relaxation equipment, visual and auditory beauties along with training of mindfulness and self-soothing skills by expert psychologists and Social support would relieve staff distress. Strengthen the meaning in work and spiritual and religious coping styles, encourage and highlight positive and effective personality traits such as commitment, hope, optimism, kindness, patience in nurses. Social support by the people, the media and managers for front-line staff in the fight against the Corona virus should not diminish during a pandemic. This support should be increased in parallel with staff burnout and fatigue.

### Limitations

This study has several limitations. Due to the health care quarantine in COVID-19 outbreak, all participants were selected from Tehran Province. Moreover, study could only explore the psychological distress at the first two weeks of the corona virus outbreak without the longitudinal observation. Finally, in addition to the factors concerned in this study, there may be other factors that affect the psychological distress. Consequently, future research can expand the region and increase the sample size.

## Conclusions

The results of this study found that there are some barriers and challenges to medical personnel exposed to COVID-19that caused psychological distress. Some of these problems related to the nature of illness, others related to social and organizational demands and some of supportive resources buffer the relationship between occupational demands and psychological distress. We discuss these factors in this article.

## Data Availability

Readers who wish to gain access to the data can write to the Corresponding author azizi.m411@gmail.com to request access.

## Abbreviation

JD-R model: Job Demands-Resources model
